# Acute ankle injuries: association between sprain severity and ancillary findings

**DOI:** 10.31744/einstein_journal/2023AO0162

**Published:** 2023-09-15

**Authors:** Frederico Celestino Miranda, Eduardo Noda Kihara, Marcelo Pires Prado, Laercio Alberto Rosemberg, Durval do Carmo Barros Santos, Atul Kumar Taneja

**Affiliations:** 1 Hospital Israelita Albert Einstein São Paulo SP Brazil Hospital Israelita Albert Einstein, São Paulo, SP, Brazil.; 2 Musculoskeletal Radiology Division Department of Radiology UT Southwestern Medical Center Dallas TX USA Musculoskeletal Radiology Division, Department of Radiology, UT Southwestern Medical Center, Dallas, TX, USA.

**Keywords:** Ankle injuries, Fractures, avulsion, Sprains and strains, Ligaments/ injuries, Magnetic resonance imaging

## Abstract

**Objective:**

To correlate the significance of osseous, chondral, tendon, and ligamentous injuries with anatomical variations in low-grade *versus* high-grade acute ankle sprains.

**Methods:**

We retrospectively identified the magnetic resonance imaging findings of acute ankle sprains (<15 days). Participants with a history of previous sprains, arthritis, tumors, infections, or inflammatory conditions were excluded. Images were independently evaluated by two musculoskeletal radiologists and assessed for osseous, chondral, tendon, and ligamentous injuries and anatomical variations. Participants were divided into low-grade versus high-grade sprain groups, according to the presence of a complete tear in at least one component of the lateral ligament complex.

**Results:**

The final study group comprised 100 magnetic resonance images (mean age, 36 years), the majority of males (54%), the right ankle (52%), and a mean sprain duration of 5 days. Participants with high-grade sprains presented with increased rates of medial malleolus edema (p<0.001), moderate and large articular effusions (p=0.041), and shorter calcaneonavicular distance (p=0.008). Complete tears of the anterior talofibular ligament and calcaneofibular ligaments were observed in 100% and 51.2% of the participants in the High-Grade Group, respectively. The deltoid ligament complex was partially torn in this group (55.8% *versus* 8.8%, p<0.001). Extensor tendon retinaculum lesions occurred significantly more frequently in this group (41.9%) compared to the overall study population (23%) (p<0.001).

**Conclusion:**

Participants with high-grade ankle sprains presented with shorter calcaneonavicular distances and increased rates of medial malleolus edema, deltoid complex partial tears, extensor retinaculum lesions, and articular effusion.

## In Brief

Miranda et al. reported a correlation between the significance of injuries to osseous, chondral, tendon, and ligamentous tissues in participants with low-grade versus high-grade acute ankle sprains. They demonstrated that participants with high-grade ankle sprains presented with shorter calcaneonavicular distances and increased rates of structural abnormalities compared to those with low-grade sprains. Special attention should be paid to acute ankle sprains in emergency settings to avoid failure in detecting severe injuries that could lead to chronic pain, impairment, or instability.

## Highlights

Participants presenting acute ankle sprains (<15 days) were divided into low-grade versus high-grade sprain,according to the presence of a complete tear in at least one component of lateral ligament complex.

High-grade ankle sprains group presented increased rates of medial malleolus bone bruise, deltoid ligament tears,extensor retinaculum lesions, and articular effusion.

The calcaneonavicular distance was statistically shorter in patients with high-grade sprains (median, 3.0mm) when compared to those with low-grade sprains (median, 4.0mm)

## Graphical abstract

**Figure:**
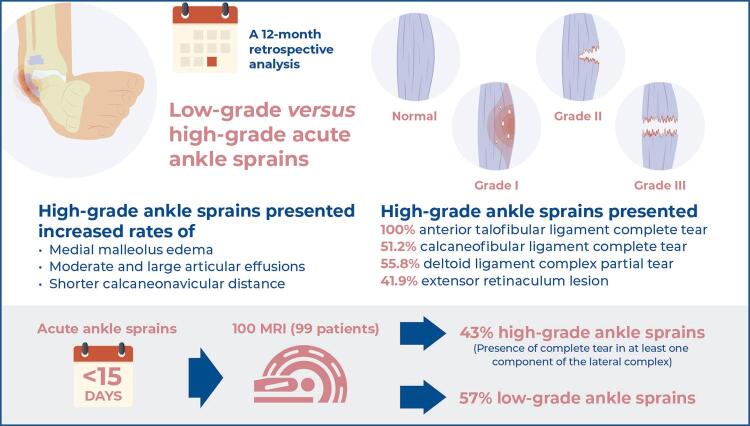

## INTRODUCTION

Ankle fractures and sprains are extremely common injuries, among the top reasons for medical care in emergency services and orthopedic clinics. They are commonly related to the inversion and adduction of the plantar-flexed foot.^(1-4)^ Sprains can affect young and professionally active individuals involved in regular physical activities and may lead to serious personal and economic consequences.^(5)^

Given the frequency of ankle ligament injuries, structural abnormalities in patients with chronic instability are well documented,^(6,7)^ but there are few large MRI studies correlating structural injuries due to acute ankle sprains.^(4,8,9)^

The anterior talofibular ligament is affected in most torsional traumas, occurring in up to 90% of major ankle injuries; the calcaneofibular ligament is involved in 50–75% of cases, and the posterior talofibular ligament is involved in 10%.^(4,10)^Tibiofibular ligament injuries occur in 1–18% of cases.^(4)^ In addition to ligament injuries, other associated injuries include osteochondral fractures of the talar dome, fractures of the lateral process of the talus, fractures of the medial or lateral tubercle of the posterior process of the talus, fractures of the anterior process of the calcaneus and injuries of the bifurcate ligament.

The literature indicates that a routine ankle magnetic resonance imaging (MRI) protocol has a wide range of specificity (70–97%) and sensitivity (40–95%) for the diagnosis of acute anterior talofibular ligament injury.^(10,11)^ Most of the literature has focused primarily on chronic stability, and there is a lack of data on the prevalence of injuries associated with severe acute torsional trauma. Moreover, MRI is not routinely requested for acute ankle sprains because the reference standard for the diagnosis of ankle sprains is a physical examination combined with a radiographic assessment.^(4,8-10)^

## OBJECTIVE

To investigate the correlation between the severity of injuries to osseous, chondral, tendon, and ligamentous tissues in individuals with low-grade *versus* high-grade acute ankle sprains, as well as to establish a relationship between anatomic features and the severity of the sprain.

## METHODS

Selection of participants and clinical information.

A retrospective analysis was performed for ankle MRI of patients with acute ankle sprains, with <15 days between the date of injury and the MRI examination performed at our institution from January to December 2014, regardless of age or sex. The exclusion criteria included history or imaging findings of a previous sprain, arthritis, tumors, infections, or inflammatory conditions affecting the ankle. Data on the participants’ clinical profiles and the time of ankle sprain were recorded.

### Imaging studies

All exams were performed following the departmental protocol, either 1.5 T or 3.0 T MRI scanners, in the supine and neutral positioning of the ankle in a dedicated phased array coil. The majority of the exams were performed under the following sequences parameters: sagittal T2 fat suppresed (Time of Repetition, TR, 3090, Time of Echo, TE, 40; FOV, Field of View, 15cm; slice thickness, 3.5mm; matrix, 320 x 224; NEX, number of excitations, 3), sagittal T1 (TR, 402, TE, 7.7; FOV, 15cm; slice thickness, 3.5mm; matrix, 512 x 256; NEX, 2), coronal T2 Fat Suppresed (TR, 3950, TE, 40; FOV, 15cm; slice thickness, 3.5mm; matrix, 320 x 224; NEX, 2), coronal oblique PD–proton density (TR, 2670, TE, 44; FOV, 15cm; slice thickness, 3.5mm; matrix, 384 x 256; NEX, 3), axial T1 (TR, 605, TE, 7.7; FOV, 15cm; slice thickness, 3.5mm; matrix, 320 x 256; NEX, 1), axial T2 Fat Suppresed (TR, 4450, TE, 40; FOV, 15cm; slice thickness, 3.5mm; matrix, 320 x 224; NEX, 2). Intravenous contrast-enhanced pulse sequences were performed in some participants according to clinical indications; however, these were not used for analysis.

### Reading method and qualitative parameters

All MRI examinations were independently evaluated by two fellowship-trained musculoskeletal radiologists (with 7 years of experience [reader 1] and 5 years of experience [reader 2]) and assessed for osseous, chondral, tendon, and ligamentous abnormalities using all three planes on picture archiving and communications system (PACS). The readers were blinded to the patient reports and clinical information.

The following anatomical structures and findings were assessed in each case by both readers:

The lateral ligament complex of the ankle was graded as normal, sprained, partial tear, complete tear, or scarred. The anterior talofibular, posterior talofibular, and calcaneofibular ligaments were evaluated separately. During the analysis, the participants were categorized into two groups based on the severity of their ankle sprains: low-grade or high-grade, depending on whether they had a complete tear in at least one component of the lateral complex.

Syndesmotic ligaments (anterior tibiofibular ligament, posterior tibiofibular ligament, transverse ligament, and interosseous membrane) were evaluated similarly to the lateral ligaments.

The deltoid ligament was divided into superficial and deep components and graded as a normal, sprained, partial tear, or complete tear.

Spring ligament was graded as normal or pathologic.

Bony structures were evaluated and graded for contusions, fracture/avulsions, accessory ossicles, and tarsal coalitions. The locations of these changes were recorded. Osteochondral lesions were also evaluated using the Anderson Classification, and their locations were annotated.

Tendons (Achilles, posterior tibialis, flexor digitorum longus, flexor hallucis longus, peroneus longus, and peroneus brevis) were evaluated and classified as normal or pathological. The flexor, peroneal, and extensor retinacula were classified as normal, sprained, or torn.

Sinus tarsi were evaluated and classified as normal or pathologic.

Articular effusion was assessed and classified as physiological/minimum when a small amount of fluid was present in the posterior tibiotalar and subtalar recesses. Joint effusion was considered present with articular capsular distension and was graded as small (either anterior or posterior capsular distension), moderate (both anterior and posterior capsular distension), or large (anterior, posterior, and lateral capsular distension).

The plantar fascia was graded as normal, fasciopathy without edema, or fasciopathy with edema.

Calcaneonavicular distance was measured in millimeters (mm) using a sagittal T1-weighted image that included the tip of the anterior calcaneal process and the proximal lateral border of the navicular bone, using the cortical bone as a reference.

This study was approved by the Ethics Committee of *Hospital Israelita Albert Einstein* (CAAE: 45351315.1.0000.0071; #1.150.359), with an exemption status for individual informed consent.

### Statistical analyses

The profiles of the participants included in the study were described as absolute and relative frequencies for categorical parameters and as quartiles for numeric parameters. They were correlated to the severity of the sprain using χ^2^ and Mann–Whitney tests. The distribution of the numerical variables was verified using boxplots.

The reliability of the assessed data was measured by interobserver agreement, which was conducted to identify lesions, fractures, and the severity of lesions but not to specify the location or type of the lesion. For this evaluation, we used the AC coefficient between the readers, followed by a confidence interval of 95% (95%CI) and p-values.^(12)^Ordinal weights were used to assess the severity. Particularly for the calcaneonavicular sagittal distance, the intraclass correlation coefficient (ICC) and Bland-Altman graphs were analyzed.^(13)^ Intraclass correlation coefficient values interpretation followed levels proposed by Altman, being classified as poor (<0.2), fair (0.2–0.4), moderate (0.4–0.6), good (0.6–0.8), and excellent (>0.8).^(14)^

The prevalence of findings was described as absolute or relative frequencies and was correlated with the severity of the sprain using χ^2^ or Fisher’s exact Student’s *t*-test, depending on data adequacy. To compare the location or type of lesions and correlate them with severity, we used Fisher’s exact Student’s *t*-test with Hommel’s method-adjusted p-values, considering the total number of locations for each measurement.^(15,16)^Such adjustments were required because the same participant may present with more than one type of lesion or location. Ordinal or numerical measures were compared using the Mann–Whitney U test. The distribution of numerical values was confirmed using boxplots or the Shapiro–Wilk normality test.

Statistical analyses were performed using R v3.1.3 (R Core Team), and the significance level was set at 5%.

## RESULTS

### Participant selection

The final study group comprised 100 consecutive ankle MRIs from 99 distinct participants who underwent ankle MRI presenting with a history of acute ankle sprain (in <15 days), following the exclusion criteria mentioned in the materials and methods.

### Clinical information and imaging studies

The mean age was 36.0 years (range, 8–81 years). There were 46% women (46/100) and 54% men (54/100). The left ankle was scanned in 48% (48/100) of patients, and the right ankle in 52% (52/100). Ankle sprains occurred between 0 and 14 days, with a mean duration of 5 days. Three participants experienced sprains on the same day of the MRI examination.

### Reading parameters and statistical analyses

The participants were divided into high- and low-grade ankle sprains. Ankle sprains were classified as high grade when at least one of the components of the lateral ligamentous complex (anterior talofibular, posterior talofibular, and calcaneofibular ligaments) had a complete tear.

There were no significant differences between the groups regarding sex (54% male, p=0.083), age (36 years old, median, p=0.802), the affected side (52% right ankle) p=0.119), and mean time since the sprain (5 days, median, p=0.989). The characteristics of the study groups are presented in table 1.

**Table 1:** Study group characteristics

	Total	Sprain		p value
		High-Grade Sprain Group	Low-Grade Sprain Group	
Number	100	43	57	
Female, (%)	46	34.9	54.4	0.083
Male, (%)	54	65.1	45.6	
Age (years)	36	35	36	0.802
Right ankle, (%)	52	41.9	59.6	0.119
Left ankle, (%)	48	58.1	40.4	
Time of sprain (days)	5	5	5	0.989

Intraclass correlation coefficient ranged from moderate to perfect between readers (ICC = 0.42–1.00) for lesion identification. From a total of 18 items evaluated, 14 (77.8%) of them showed excellent agreement between the readers (ICC >0.8), two of them showed good agreement (ICC = 0.6–0.8), and in two of them, agreement was moderate (ICC = 0.4–0.6), which were: posterior tibial tendon (ICC = 0.42) and Achilles tendon (ICC = 0.54) (Table 2). Regarding injury classification, readers had an excellent agreement in 12 of the evaluated parameters, with ICC varying from 0.81 to 0.99 (Table 3). The ICC between the readers for calcaneonavicular distance in the sagittal plane was 0.81, indicating excellent agreement. The Bland–Altman graph showed no trend in the differences observed between the two measurements.

**Table 2:** Readers’ agreement on lesion identification

Parameter	ICC	95%CI	p value
Bone bruise	0.91	0.83; 0.98	<0.001
Fracture and avulsion	0.92	0.84; 0.99	<0.001
Accessory bones	1.00	-	-
Coalition	1.00	-	-
Loose bodies	1.00	-	-
Deltoid ligament	0.91	0.85; 0.97	<0.001
Spring ligament	0.91	0.85; 0.98	<0.001
Calcaneal tendon	0.54	0.37; 0.70	<0.001
Posterior tibial tendon	0.42	0.24; 0.60	<0.001
Flexor digitorum longus tendon	0.97	0.93; 1.00	<0.001
Flexor hallucis longus tendon	0.99	0.97; 1.00	<0.001
Peroneus longus tendon	0.60	0.44; 0.76	<0.001
Peroneus brevis tendon	0.69	0.54; 0.83	<0.001
Flexor retinaculum	0.99	0.97; 1.00	<0.001
Peroneal	1.00	-	-
Tarsal sinus	0.99	0.97; 1.00	<0.001
Accessory muscle/tendon	1.00	-	-
Soft tissue edema	1.00	-	-

ICC: intraclass correlation coefficient; 95%CI: 95% confidence interval.

**Table 3:** Readers’ agreement on injury classification

Parameter	ICC	95%CI	p value
Osteochondral lesion	0.99	0.98; 1.00	<0.001
Joint effusion	0.98	0.97; 1.00	<0.001
Anterior talofibular ligament	0.96	0.91; 1.00	<0.001
Posterior talofibular ligament	0.85	0.78; 0.92	<0.001
Fibulocalcaneal ligament	0.92	0.86; 0.99	<0.001
Anterior tibiofibular ligament	0.93	0.86; 0.99	<0.001
Posterior tibiofibular ligament	0.98	0.96; 1.00	<0.001
Interosseous ligament	0.99	0.98; 1.00	<0.001
Deltoid ligament	0.99	0.98; 1.00	<0.001
Extensor retinaculum	0.81	0.70; 0.93	<0.001
Plantar fascia	0.96	0.91; 1.00	<0.001
Calcaneonavicular distance	0.81	0.73; 0.87	<0.001

ICC: intraclass correlation coefficient; 95%CI: 95% confidence interval.

### Qualitative parameters

**Figure 1:**
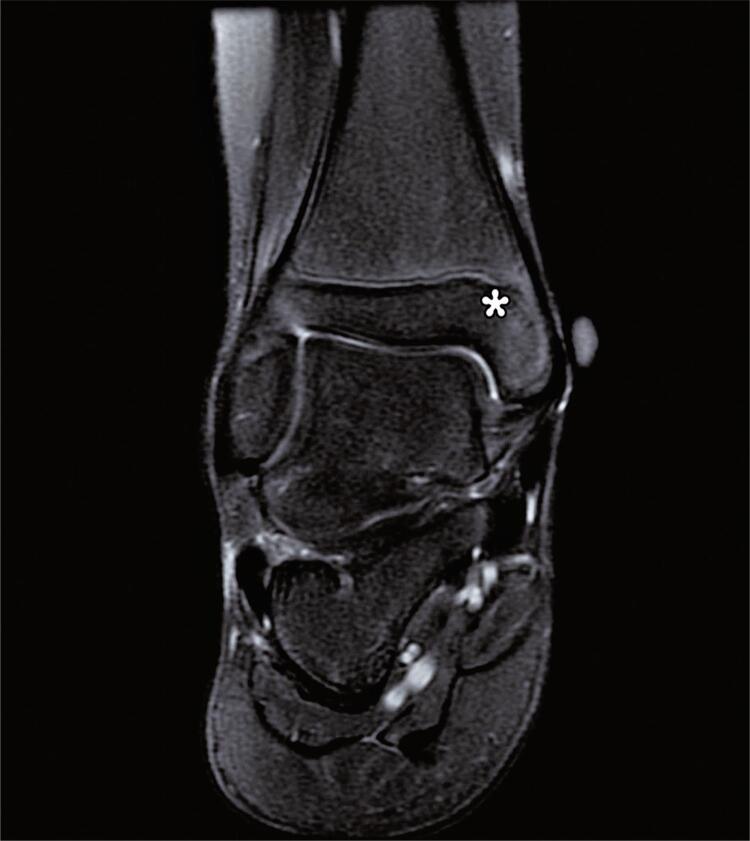
Coronal T2-weighted fat-suppressed magnetic resonance image shows bone bruise at the medial malleolus (star)

The most common ancillary injury was a bone contusion, occurring in 76% of the ankles, with the most prevalent site being the talus in 35% of the ankles. A statistically significant difference was observed between high- and low-grade sprains in relation to bone edema in the medial malleolus (Figure 1), with a greater proportion of high-grade sprains (44.2%).

Fractures and/or avulsions were present in 27% of cases, most commonly at the distal fibula and anterior talofibular ligament insertion site (11%). Accessory bones were observed in 29% of cases, with 16% being accessory navicular bones. There were two cases of calcaneonavicular coalitions.

**Figure 2:**
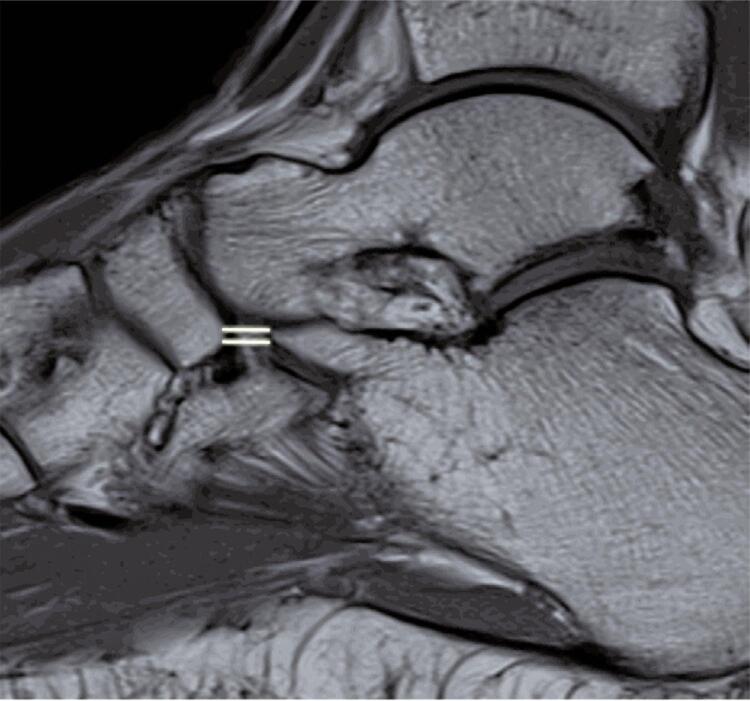
Calcaneonavicular distance was measured using a sagittal T1-weighted image that includes the tip of the anterior calcaneal process and the proximal lateral pole of the navicular (double line)

The calcaneonavicular distance was smaller in patients with severe sprains (median, 3.0mm) in comparison to patients with non-severe sprains (median, 4.0mm) (p=0.008) (Figure 2, Table 2).

Acute osteochondral lesions of the talus were observed in 19% of the cases and were graded according to Anderson’s classification^(2)^ as grade I (14%), grade II (2%), and grade III (3%). These lesions were most frequent in the posterolateral region of the talus (44.4%). More acute osteochondral lesions (grades I to III) occurred in the High-Grade Sprain Group (11%) than in the Low-Grade Sprain Group (8%).

**Figure 3:**
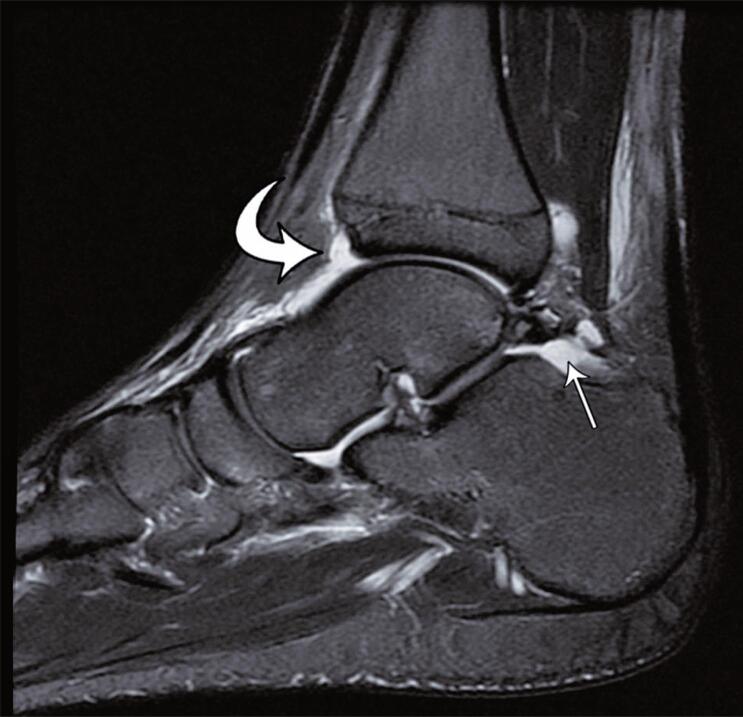
Sagital T2-weighted fat-suppressed magnetic resonance image demonstrating moderate effusion in tibiotalar (curved arrow) and subtalar joints (thin arrow)

Most joint effusions were small (52%); however, in high-grade sprains, there were higher proportions of moderate (Figure 3) and severe effusions than in low-grade sprains (p=0.041).

**Figure 4:**
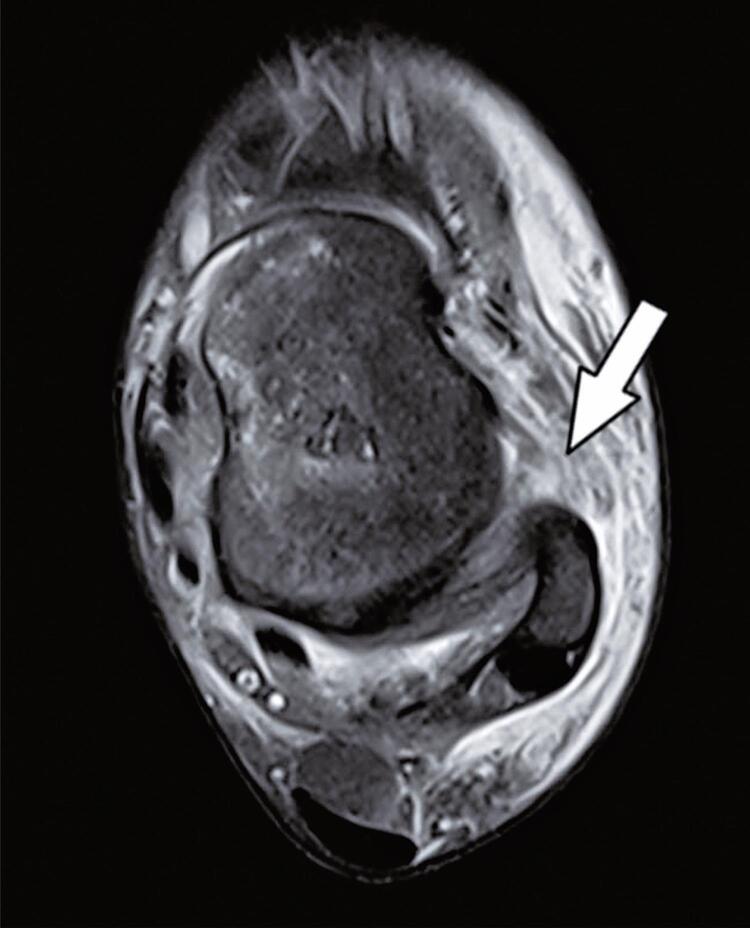
Axial T2-weighted fat-suppressed magnetic resonance image shows a complete tear of the anterior talofibular ligament (arrow). Extensive lateral ankle soft tissue edema and swelling are also seen

Complete tears affecting the anterior talofibular (Figure 4) and calcaneofibular ligaments were observed in 100% and 51.2% of the participants in the High-Grade Sprain Group, respectively.

**Figure 5:**
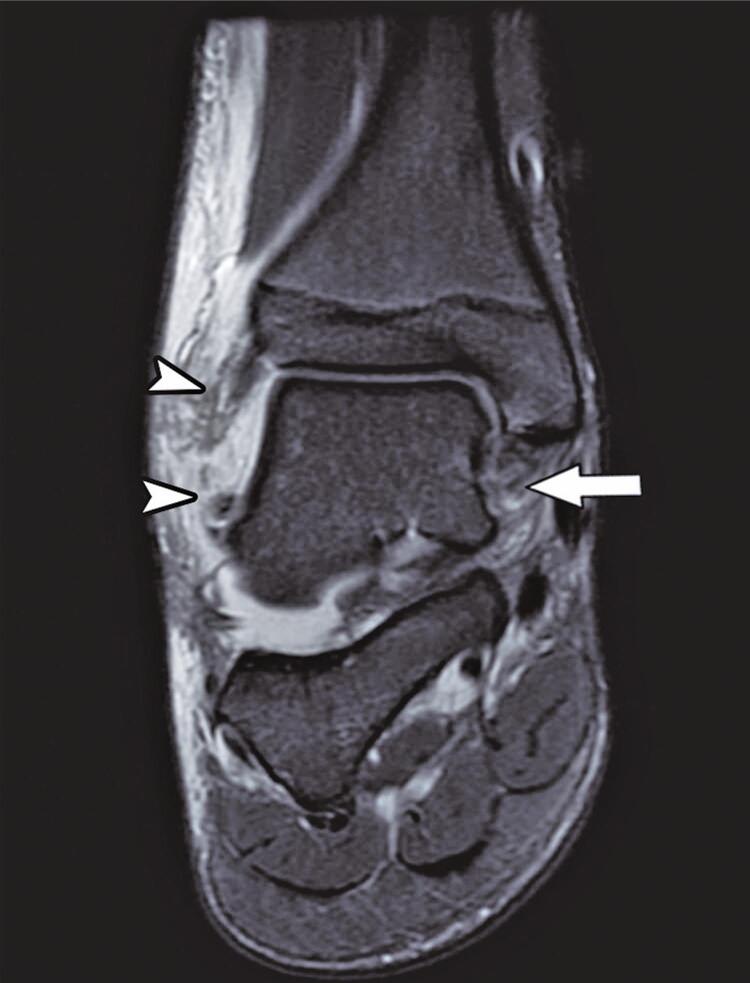
Coronal T2-weighted fat-suppressed magnetic resonance image presents partial tear of the deltoid ligament (arrow) and complete tear of the lateral ligament complex (arrowheads, partially seen on this image). Extensive lateral ankle soft tissue edema and subtalar joint effusion are also depicted

**Figure 6:**
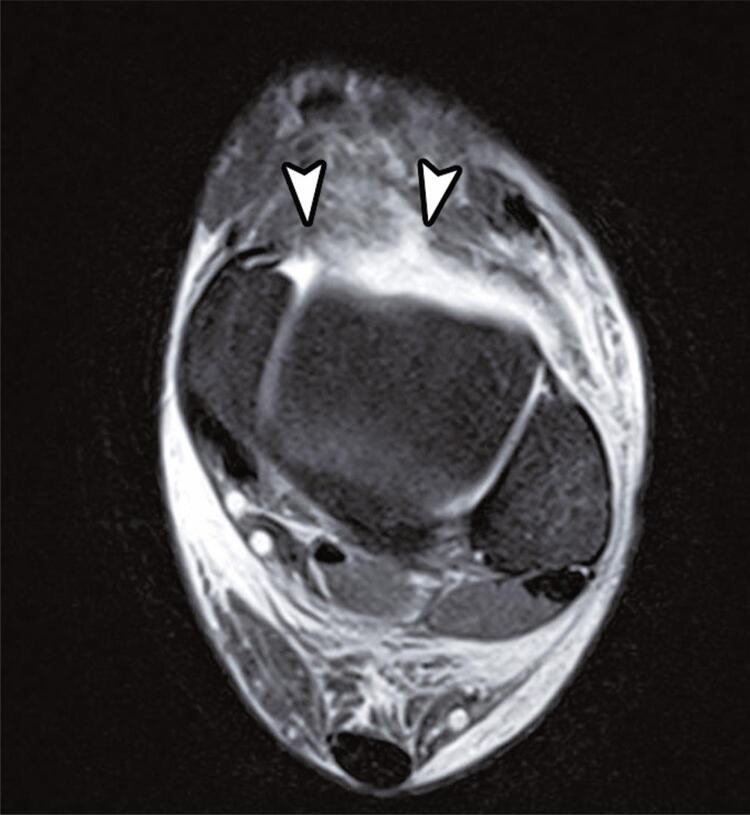
Axial T2-weighted fat-suppressed magnetic resonance image shows extensive edema involving the extensor retinaculum, along with areas of discontinuity of the fibers (arrowheads)

The deep component of the deltoid ligament was partially torn (Figure 5) in high-grade ankle sprains (55.8% *versus* 8.8%, p<0.001). Extensor retinaculum lesions (Figure 6) occurred in 23% of the patients, reaching up to 41.9% in this group (p<0.001).

All soft tissue edema findings were greater in the High-Grade Sprain Group, with statistically significant differences observed in the lateral perimalleolar region (p=0.004) and Kager’s fat pad (p=0.004).

Table 4 summarizes the main parameters with statistical correlations (p<0.05). The remaining findings did not demonstrate a significant difference between the groups (p>0.05).

**Table 4:** Main parameters with statistical correlation

	Total (100%)	High-grade (43%)	Low-grade (57%)	p value
Calcaneonavicular distance, (mm)	4.00 (3;5)	3.00 (2.5;4)	4.00 (3;5)	0.008
Medial malleolus bone edema, (%)	23	19	4	<0.001
Moderate/Large articular effusion, (%)	30	15	15	0.041
Deltoid ligament deep component (Parcial Tear), (%)	29	24	5	<0.001
Extensor retinaculum lesion, (%)	23	18	5	<0.001

Calcaneonavicular distance is presented as median (1^st^ and 3^rd^ quartiles) and the remaining parameters as percentages (frequency).

## DISCUSSION

Ankle sprains are extremely common injuries and are among the most common reasons for medical care in emergency services and orthopedic clinics.^(1)^These injuries are usually associated with torsional ankle trauma and are generally related to the inversion and adduction of the plantar-flexed foot.^(2-4)^

Rosello et al. reported that the normal distance between the anterior process of the calcaneus and the navicular bone is between 5 and 10mm, with a distance of less than 5mm on MRI, strongly indicating an enlarged anterior calcaneal process.^(17)^ Wartelle et al. reported that calcaneonavicular impingement could happen when the distance is less than 5mm, causing excessive mechanical stress on adjacent joints, leading to recurrent sprains and development of osteochondral lesions of the talar dome.^(18)^ Cavalier et al. demonstrated an association between the elongated anterior process of the calcaneus and osteochondral lesions of the talus in 76% of patients.^(19)^ Our data confirmed that participants with high-grade ankle sprains have shorter calcaneonavicular distances with a median of 3.0mm.

The calcaneonavicular distance was slightly greater in patients with low-grade sprains (median, 4.0mm) in comparison to patients with high-grade sprains (median, 3.0mm) (p=0.008). We hypothesize that a shorter calcaneonavicular distance may be related to a decreased rearfoot motion, acting as a “functional coalition,” overloading the ankle joint and increasing the chance of a sprain or its severity. In addition, we believe that these patients may have the worst prognosis, with an increased chance of poor results after conservative treatment; however, further studies are needed to confirm these hypotheses.

These patients also presented with an increased prevalence of medial malleolus bone bruises, complete talofibular and calcaneofibular ligament tears, deltoid complex partial tears, extensor retinaculum lesions, and greater articular effusions. To our knowledge, this is the first study to correlate the calcaneonavicular distance with the severity of ankle sprains.

According to Roemer et al., in a study of athletes, Barini et al. reported that at least one component of the lateral ligament complex of the ankle is affected in up to 90% of major ankle injuries, with 39.5% of complete injuries being to the anterior talofibular ligament.^(4,10)^ In our study, although not restricted to athletes, we observed similar results, with a frequency of 43% of complete tears affecting the anterior talofibular ligament, which was present in 100% of high-grade sprains. In addition, the high-grade ankle sprain group presented with a complete tear affecting the calcaneofibular ligament at a frequency of 51.2%.

In addition to ligament injuries, other associated injuries included osteochondral lesions of the talar dome, fractures, and bone edema. Given the frequency of such injuries, structural abnormalities in patients with chronic instability have been well-documented,^(6,7)^ but there are only a few MRI studies correlating structural injuries due to acute ankle sprains, with a lack of data on the real prevalence of these and other associated injuries since MRI is not routinely widely requested for acute ankle sprains. The standard approach for diagnosing ankle sprains in an acute setting is a physical examination combined with a radiographic assessment.^(4,8,9)^In our institution, a tertiary and complex hospital with a high volume of orthopedic patients in the MRI department, we often deal with acute cases, allowing us to study a cohort with this profile.

Roemer et al. demonstrated that the most common ancillary finding in athletes with acute ankle sprains was bone contusions, particularly those affecting the talus (44%).^(4)^ Our results in the general population were similar, with bone contusions in the talus being present in 35% of the patients, especially at the talar head and neck.

Acute osteochondral lesions of the talus were seen in 8% of athletes by Roemer et al.^(4)^We observed a higher prevalence in our study, reaching 19%. In the literature, the reported incidence of osteochondral lesions of the talus is based on limited case series, with studies indicating an incidence of 10% for bilateral osteochondral lesions of the talus.^(2,20,21)^ This low incidence could be explained by osteochondral lesions of the talus following an ankle sprain is likely to remain undetected or misdiagnosed as another ankle pathology.^(22,23)^ Reports have concluded that osteochondral lesions of the talus, especially lateral lesions, are primarily of traumatic origin, whereas medial lesions associated with trauma can vary from 25–80%.^(24,25)^Other reports have indicated that osteochondral lesions of the talus may have a higher incidence in patients with chronic ankle pain, probably confirming a delayed diagnosis.^(21,26)^

We found fractures and/or avulsions in 27% of the ankles, most commonly in the distal fibula (11%). Roemer et al. did not find any fractures in an athletic population, which can be explained by the fact that patients with fractures in their cohort were not referred for MRI.^(4)^

Van Putte-Katier et al. demonstrated that a substantial number of MRI abnormalities could be found in both injured and contralateral (asymptomatic) ankles after ankle sprains, especially bone edema and ATFL tears, highlighting the danger of making clinical decisions solely based on MRI without clinical correlation.^(27)^

Crema et al. demonstrated that tibiotalar and talocalcaneal effusions are associated with an increased risk of severe concomitant structural injuries in acute ankle sprains.^(8)^Most joint effusions in our study were small (52%); however, in high-grade sprains, there were higher proportions of moderate (32.6%) and severe (2.3%) effusions than in the Low-Grade Sprain Group.

The main limitations of this study are its retrospective nature, unavailability of longitudinal follow-up, and correlation between imaging and surgical findings. In addition, although injury mechanism is an important factor influencing sprain severity, such details were not available in all patient’s self-reports during MRI scheduling.

## CONCLUSION

In conclusion, our study results demonstrated that participants with high-grade ankle sprains presented with shorter calcaneonavicular distances and increased rates of structural abnormalities than those with low-grade sprains. Moreover, these rates were higher than those reported in previous studies. Special attention should be paid to acute ankle sprains in emergency settings to avoid failure in detecting severe injuries that could lead to chronic pain, impairment, or instability.

## References

[1] Baumhauer JF, Alosa DM, Renström AF, Trevino S, Beynnon B (1995). A prospective study of ankle injury risk factors. Am J Sports Med.

[2] Beynnon BD, Vacek PM, Murphy D, Alosa D, Paller D (2005). First-time inversion ankle ligament trauma: the effects of sex, level of competition, and sport on the incidence of injury. Am J Sports Med.

[3] Pijnenburg AC, Van Dijk CN, Bossuyt PM, Marti RK (2000). Treatment of ruptures of the lateral ankle ligaments: a meta-analysis. J Bone Joint Surg Am.

[4] Roemer FW, Jomaah N, Niu J, Almusa E, Roger B, D’Hooghe P (2014). Ligamentous injuries and the risk of associated tissue damage in acute ankle sprains in athletes: a cross-sectional MRI study. Am J Sports Med.

[5] Ekstrand J, Gillquist J (1983). Soccer injuries and their mechanisms: a prospective study. Med Sci Sports Exerc.

[6] Park HJ, Cha SD, Kim SS, Rho MH, Kwag HJ, Park NH (2012). Accuracy of MRI findings in chronic lateral ankle ligament injury: comparison with surgical findings. Clin Radiol.

[7] van Ochten JM, Mos MC, van Putte-Katier N, Oei EH, Bindels PJ, Bierma-Zeinstra SM (2014). Structural abnormalities and persistent complaints after an ankle sprain are not associated: an observational case control study in primary care. Br J Gen Pract.

[8] Crema MD, Krivokapic B, Guermazi A, Gravilovic P, Popovic N, D’Hooghe P (2019). MRI of ankle sprain: the association between joint effusion and structural injury severity in a large cohort of athletes. Eur Radiol.

[9] Langner I, Frank M, Kuehn JP, Hinz P, Ekkernkamp A, Hosten N (2011). Acute inversion injury of the ankle without radiological abnormalities: assessment with high-field MR imaging and correlation of findings with clinical outcome. Skeletal Radiol.

[10] Barini M, Zagaria D, Licandro D, Pansini S, Airoldi C, Leigheb M (2021). Magnetic resonance accuracy in the diagnosis of anterior talo-fibular ligament acute injury: a systematic review and meta-analysis. Diagnostics (Basel).

[11] Salat P, Le V, Veljkovic A, Cresswell ME (2018). Imaging in foot and ankle instability. Foot Ankle Clin.

[12] Gwet KL (2014). Manual de confiabilidade entre avaliadores.

[13] Hermanson E, Ferkel RD (2009). Bilateral osteochondral lesions of the talus. Foot Ankle Int.

[14] Altman DG (1990). Practical Statistics for Medical Research.

[15] Hommel G (1988). A stagewise rejective multiple test procedure based on a modified bonferroni test. Biometrika.

[16] Wright SP (1992). Adjusted p-values for simultaneous inference. Biometrics.

[17] Rosello O, Solla F, Oborocianu I, Chau E, Yagoubi F, Clément JL (2016). Too-long calcaneal process: Results of surgical treatment and prognostic factors. Orthop Traumatol Surg Res.

[18] Wartelle J, Hocquet B, Lucchesi G, Coursier R, Boutry N, Budzik JF (2022). The too-long anterior process and osteochondral lesion of the talus: Is there an anatomical predisposition? A case-control study on 135 feet. Foot Ankle Surg.

[19] Cavalier M, Chau E, Raux S, Solla F, El Batti S, Oborocianu I (2015). L’ostéochondrite du talus est-elle associée au bec calcanéen trop long?. Revue Chirurgie Orthopédique Traumatol.

[20] Anderson IF, Crichton KJ, Grattan-Smith T, Cooper RA, Brazier D (1989). Osteochondral fractures of the dome of the talus. J Bone Joint Surg Am.

[21] Rungprai C, Tennant JN, Gentry RD, Phisitkul P (2017). Management of Osteochondral Lesions of the Talar Dome. Open Orthop J.

[22] Benthien RA, Sullivan RJ, Aronow MS (2002). Adolescent osteochondral lesion of the talus. Ankle arthroscopy in pediatric patients. Foot Ankle Clin.

[23] Koç A, Karabiyik Ö (2018). MRI evaluation of ligaments and tendons of foot arch in talar dome osteochondral lesions. Acta Radiol.

[24] De Smet AA, Fisher DR, Burnstein MI, Graf BK, Lange RH (1990). Value of MR imaging in staging osteochondral lesions of the talus (osteochondritis dissecans): results in 14 patients. AJR Am J Roentgenol.

[25] Takao M, Ochi M, Uchio Y, Naito K, Kono T, Oae K (2003). Osteochondral lesions of the talar dome associated with trauma. Arthroscopy.

[26] Ferkel RD, Zanotti RM, Komenda GA, Sgaglione NA, Cheng MS, Applegate GR (2008). Arthroscopic treatment of chronic osteochondral lesions of the talus: long-term results. Am J Sports Med.

[27] van Putte-Katier N, van Ochten JM, van Middelkoop M, Bierma-Zeinstra SM, Oei EH (2015). Magnetic resonance imaging abnormalities after lateral ankle trauma in injured and contralateral ankles. Eur J Radiol.

